# Mr. Edlin, on Yeast

**Published:** 1801-05

**Authors:** A. Edlin

**Affiliations:** Uxbridge


### *To the Editors of the Medical and Physical Journal.*

Gentlemen,

If the following observations respecting Yeast, merit a place in your valuable Journal, you will much oblige me by inserting them next month.

*Uxbridge,*

*20th March,* 1801.

I am, &c.

A. EDLIN.

---

When professional men come forward in a candid manner to announce to the world the result of such new remedies as they have employed, either from their own suggestion or the recommendation of others, it is rather unfair to criticise their opinions and condemn their practice, without deliberate reflection, or even without a trial; instead of attempting by experiment and enquiry, to appreciate the merit of such discovery.

The very act of holding up new remedies to the shafts of ridicule, may deter timid practitioners from adopting them; hence the spirit of enquiry is stifled, and the cause of truth extinguished. In this point of view I am led to consider a controversy that has taken place between Mr. Grose and Mr. Custance, respecting the use of yeast in typhus fever.

If Mr. Custance, instead of treating the practice as empirical, and dilating upon it with levity, had examined the subject with seriousness and deliberation; if he had taken the trouble to investigate the nature and properties of yeast, and considered the effects of its constituent principles in the cure of fever, the judicious part of the profession would then have received his observations with candour; and although they might have dissented from his opinion, would have respected his motives. But when a medicine is unjustly condemned, without the least respect to practice, and in open violation of all sound reasoning, it behoves every one who has the good of his profession at heart, to step forward, and prevent, if possible, such pernicious doctrines from being implicitly believed.

I, in common with many other gentlemen, have tried and experienced the beneficial effects of yeast in typhus fever, and I trust many more will try also. I reported in a little pamphlet on malignant fever (which Mr. Grose has done me the honour to recommend), a few cases where I had found it particularly serviceable, and since that time have had frequent occasion to try it in similar complaints, and have uniformly seen it do good. I will not go so far as to assert, that yeast will effect a cure alone; yet, notwithstanding, it may be considered as a most powerful powerful auxiliary; and often when the ſtomach is ſo irritable that bark will be inſtantly rejected, a cautious exhibition of yeaſt will quiet that irritation, and keep alive the vital ſpark, till more powerful means can be had recourſe to.

It is in this way I am perſuaded, that Mr. Groſe has exhibited yeaſt, adapting that and other remedies to the exigencies of the moment, varying his plan according to the ſymptoms, and thus ſhewing himſelf a ſkilful phyſician in oppoſition to the empiric, who boaſts of his noſtrum to cure every diſeaſe.

In order to induce your readers to try ſo promiſing a remedy, I beg leave to detail the following experiment, and from the concluſions that naturally ariſe, I truſt a ſatisfactory explanation will be given, why yeaſt is ſerviceable in typhus fever.

I took a portion of yeaſt freſh from the brewhouſe, and put it into a glaſs retort; this was placed over the peumato-chemical apparatus, and by means of a lamp received over ſeveral vials of a gas which were ſubjected to teſts that clearly proved it to be carbonic acid gas, as expected.

This ſubject naturally brings to our recollection the experiments of Dr. Macbride, who was not only enabled to preſerve meat from putrefaction, by incloſing it in veſſels of fixed air, but alſo to reſtore ſuch as was bordering upon putrefaction to its original purity again; and he was of opinion that this gas united with the putrid ſubſtance, and reſtored the acid it had loſt during the time of putrefaction.

It may be argued, that a complete putrefaction is not a complaint of the human machine that can be an object of practice, becauſe it cannot take place in any important part of the body without extinguiſhing life; but there is a tendency to it in a conſiderable degree in various morbid diſorders, which yeaſt is very likely to correct or reſiſt; hence it is evident, that ſuch articles of Materia Medica as contain a large portion of fixed air, in a ſtate to be gradually evolved in the ſtomach, are of great uſe in fevers of a putrid tendency; and this accounts for the good effects of cyder, bottled porter, and yeaſt; and whatever may be its mode of action, the fact is indiſputable, that fixed air takes off that extreme debility of the ſtomach ſo conſpicuouſly marked in theſe diſorders; and in proportion as that ſubſides the pulſe riſes, becomes ſlower and fuller, the cauſtic heat on the ſkin diſappears, and a truce is gained for the reception of more nouriſhing ſupplies.

Such is my opinion concerning yeaſt as an antiſeptic, and I truſt that Mr. Cuſtance's liberality will excuſe the freedom of theſe remarks, and flatter myſelf, that at ſome future period he will alſo come forward with his teſtimony in ſupport of its credit in typhus fever.

